# Case report: A novel *CASK* mutation in a Chinese female child with microcephaly with pontine and cerebellar hypoplasia

**DOI:** 10.3389/fgene.2022.856636

**Published:** 2022-09-07

**Authors:** Guilan Xie, Yan Zhang, Wenfang Yang, Liren Yang, Ruiqi Wang, Mengmeng Xu, Landi Sun, Boxing Zhang, Xiaoyi Cui

**Affiliations:** ^1^ Department of Obstetrics and Gynecology, Maternal and Child Health Center, The First Affiliated Hospital of Xi’an Jiaotong University, Xi’an, China; ^2^ School of Public Health, Xi’an Jiaotong University Health Science Center, Xi’an, China; ^3^ Center for Translational Medicine, The First Affiliated Hospital of Xi’an Jiaotong University, Xi’an, China; ^4^ Peking University Health Science Center, Beijing, China

**Keywords:** CASK gene, whole exome sequencing, microcephaly, cerebellar hypoplasia, intellectual disability

## Abstract

**Objective:** Microcephaly with pontine and cerebellar hypoplasia (MICPCH) is a rare X-linked dominant genetic disease, and most MICPCHs are ascribed to *CASK* mutations, while few are revealed in Chinese patients. This study aims to identify the pathogenic mutation in a Chinese proband with MICPCH.

**Methods:** A 3-year-old female Chinese proband with MICPCH and her parents were included. Clinical data were collected from the medical records and recalled by the proband’s mother. Whole genome sequencing and Sanger sequencing were used to find the pathogenic mutation of MICPCH.

**Results:** The proband presented with postnatal progressive microcephaly, cerebellar hypoplasia, intellectual disability, motor and language development retardation and limb hypertonia. Genetic analysis indicated that there was a novel compound heterozygote nonsynonymous mutation, c.755T>C(p.Leu252Pro) in exon8 of *CASK* gene in the proband, but not in her parents. This *CASK* mutation has not been reported in other databases.

**Conclusion:** This study broadens the mutation spectrum of the *CASK* gene and is of great value for precise prenatal diagnosis and genetic counseling.

## Introduction

Microcephaly with pontine and cerebellar hypoplasia (MICPCH) is a rare X-linked dominant genetic disease characterized by postnatal progressive microcephaly, intellectual disability, pontine cerebellar hypoplasia, epilepsy, sensorineural deafness, and ophthalmologic abnormalities (Nishio et al., 2021; Shelby et al., 2021). Najm et al. (Najm et al., 2008) first ascribed MICPCH to calcium/calmodulin-dependent serine protein kinase (*CASK*) mutations, for they recovered heterozygous loss-of-function *CASK* mutations in individuals with MICPCH. A total of 153 mutations in the *CASK* gene have been reported in the HGMD Professional 2021.4 database, involving missense, nonsense, splicing, deletions, insertions, indels, duplications and complex rearrangements. The loss-of-function mutations in the *CASK* gene usually lead to phenotypic manifestation in females, while resulting *in utero* lethality in males (Moog et al., 2015; Mukherjee et al., 2020).

The *CASK* gene is located at Xp11.4 and encodes the *CASK* protein composed of 926 amino acids (Hackett et al., 2010). The *CASK* protein belongs to the membrane-associated guanylate kinase family and involves multiple functional domains, including L27 (LIN-2 and LIN-7 interaction), PDZ (PSD95, Discs-large, ZO-1), SH3 (src homology 3), and additional N-terminal calcium/calmodulin-dependent kinase (CaMK) and C-terminal guanylate kinase (GK) domains (Hsueh, 2009). In central nervous system synapse, *CASK* assembles multiprotein complexes and is involved in synaptic interaction, protein trafﬁcking, signaling of ion channels, and regulation of gene expression during neural development (Moog et al., 2011).

Despite the known linkages of *CASK* mutations and MICPCH, few were revealed in Chinese patients. Herein, a novel *CASK* mutation in a female Chinese child with MICPCH was reported, and it had not been reported in other databases. This finding might broaden the spectrum of the *CASK* genotype and help to precisely conduct prenatal diagnosis and genetic counseling.

## Methods

### Study objects

One female Chinese child with MICPCH and her parents were included in this study. The clinical data of the proband, comprising weight, length, head circumference at birth and different stages, progress of motor and intelligence growth, and brain magnetic resonance imaging (MRI) features, were obtained from the medical records and report from her mother. The growth status of head circumference, weight and length of the proband were compared with the growth reference standard for children in China (Department of Maternal and Child Health, National Health Commission of the People’s Republic of China, 2009). The history of diseases and medications during pregnancy, family history of related diseases and feeding status in infancy were recalled by her mother. This study was permitted by the Medical Ethics Committee of the First Affiliated Hospital of Xi’an Jiaotong University (No. XJTU1AF2021LSK-382), and written informed consent was obtained from the parents of the proband.

### Molecular analysis

After gaining the written informed consent from the parents of the proband, 2 ml venous blood samples of the proband and her parents were collected in anticoagulant tubes containing EDTA. Genomic DNA was extracted by a QIAamp DNA extraction kit (QIAGEN, Hilden, Germany), and 3 μg DNA was collected as fragments ranging from 150 bp to 200 bp with Covaris S2 (Covaris Company, United States). NEB Next DNA of Illumina (NEB Company, United States) was used to construct the whole genomic DNA library. Liquid phase capture target gene technology was used to capture the human genome gene coding exon regions, and an Illumina Nextseq 6000 (Illumina, San Diego, United States) was used to sequence the genomic DNA of the family with 150 bp pair-end sequencing mode. The low-quality data were removed after the sequencing of the target region, and the sequencing depth, uniformity and probe specificity were analyzed. Single nucleotide polymorphisms (SNPs) and InDels were analyzed with the Verita Trekker^®^ variant site detection system and the Enliven^®^ variant site annotation interpretation system (Berry Genomics, China). The SNPs and InDels were searched in the Berry big data population database, the Exome Aggregation Consortium (ExAC), the 1000 Genome Project, and the Genome Aggregation Database (gnomAD), and the variants with allele frequency lower than 5‰ were filtered. The pathogenicity of the mutation was predicted by SIFT, PolyPhen2, MutationTaster, REVEL, MCAP and LRT. Primers were synthesized and amplified by the PCR method. Sanger sequencing was conducted with an ABI3730xl sequencer (Applied Biosystems, United States), and the results were analyzed and compared with the reference sequence through Mutation Surveyor.

## Results

### Clinical features

The 3-year-old female Chinese proband was the first gravidity and the first parity of the healthy and non-consanguineous parents. The clinical and molecular characteristics of the proband are outlined in Table 1. She was delivered vaginally, with a gestational age of 41 weeks, birth weight of 3.4 kg and birth length of 51 cm. The Apgar scores were 9 points at 1 min after birth and 10 points at 5 min after birth. There was no history of asphyxia or hypoxia, and the family history of genetic diseases or microcephaly was negative. Her mother did not have gestational hypertension or gestational diabetes mellitus during pregnancy. However, at 6 months of pregnancy, the ultrasound-based gestational age was 3 weeks younger than that of the last menstrual period-based gestational age. Her mother took folate before conception but not during the pregnancy for the emesis gravidarum. Minor facial features were observed, including a round face, small chin and large ears.

**TABLE 1:** Clinical and molecular characteristics of the proband.

Variables	Characteristics
Clinical characteristics	
Age	3 years old
Gender	Female
Gestational age at birth	41 weeks
Birth weight	3.4 kg
Birth length	51 cm
Gravidity	First
Parity	First
Delivery model	Vaginal delivery
Apgar scores	
1 min after birth	9 points
5 min after birth	10 points
History of asphyxia	No
History of hypoxia	No
Family history of genetic diseases	No
Family history of microcephaly	No
Microcephaly	Yes
Pontine and cerebellar hypoplasia	Yes
Intellectual disability	Yes
Developmental delay	Yes
Facial feature	Round face, small chin and large ears
Muscular tone	Normal axial tone and limb hypertonia
Seizures	No
Hearing loss	No
Ophthalmologic abnormalities	No
Molecular characteristics	
Position	exon8
Nucleotide change	c.755T>C
Amino-acid change	p.Leu252Pro

**FIGURE 1:**
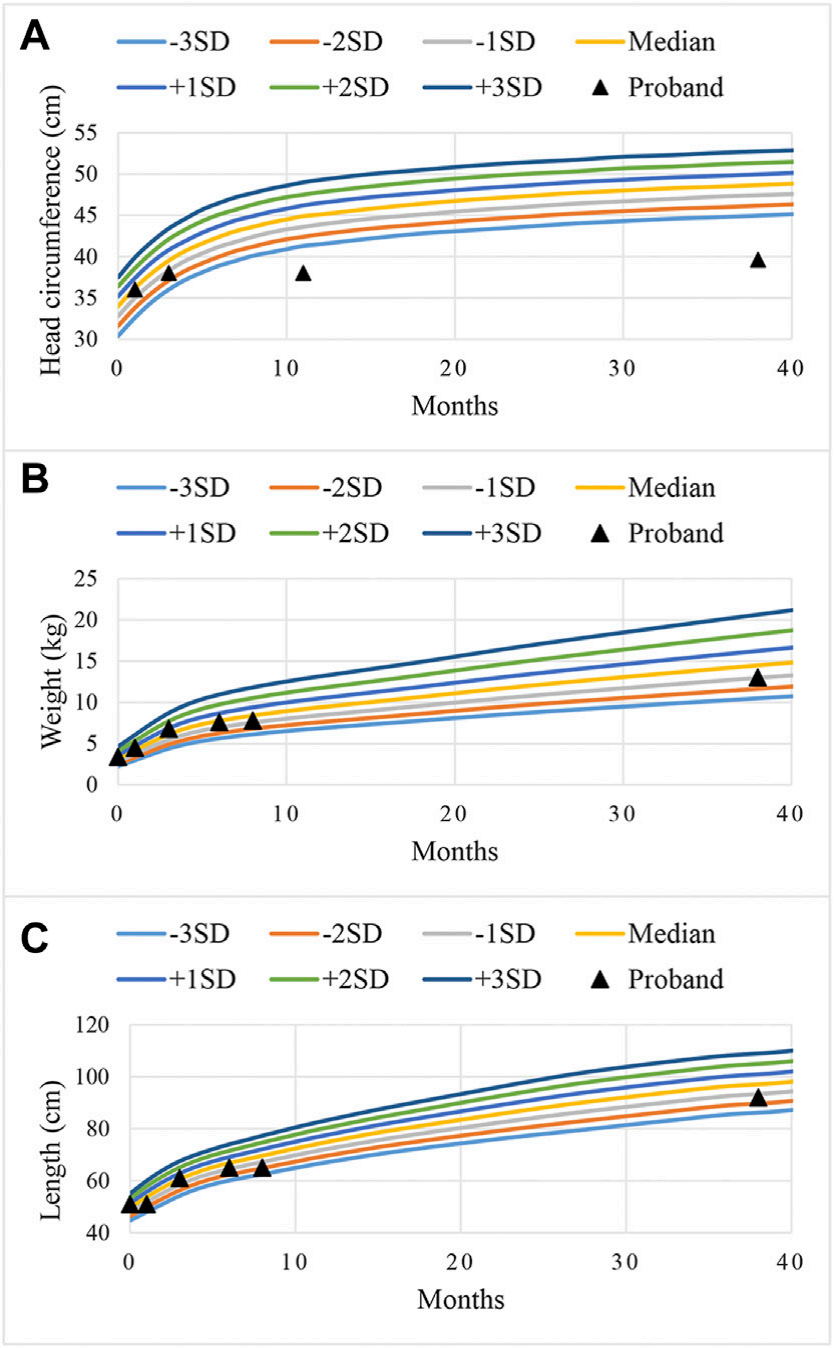
Curves of standard deviations (SD) during the growth of head circumference **(A)**, weight **(B)**, and length **(C)** for the proband. The color curves were the referenced growth curves for Chinese girls, and the black triangles were the growth status of the proband at different ages.

**FIGURE 2:**
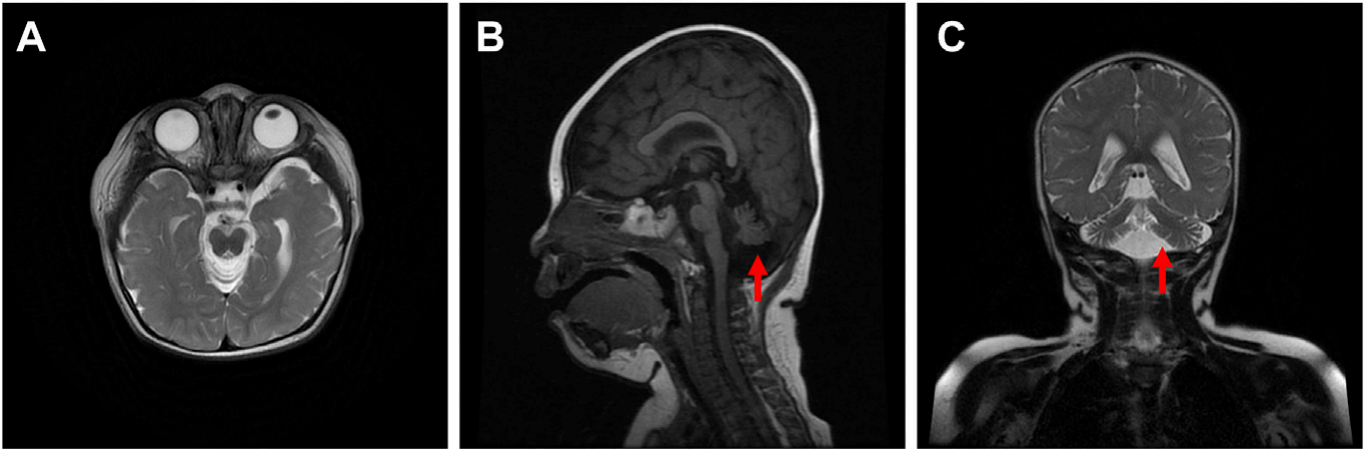
Axial **(A)**, sagittal **(B)**, and coronal **(C)** brain magnetic resonance images of the proband.

The proband was diagnosed with postnatal progressive microcephaly. The growth curves of head circumference, weight and length for the proband are shown in Figure 1. The head circumference was initially within the normal range, but the growth of head circumference nearly came to a standstill at 3 months. Her weight and length were always in the normal range for Chinese girls, although sometimes at the low-normal level. In infancy, she had poor swallowing function, with drooling and feeding difficulties. She also had an intellectual disability and delayed motor development. The results of Gesell Developmental Schedules indicated that her status of adaptive, gross motor, fine motor, language and personal-social behaviors were delayed. When she was at the age of 11 months old, her status matched the age of 20–26 weeks. The brain magnetic resonance imaging (MRI) presented with cerebellar hypoplasia and bilateral enlarged brain ventricles (Figure 2). For the muscle tone, the axial tone was normal but with limb hypertonia. At the age of 3 years, she could only turn over, sit unaided, and crawl with abnormal posture. She could not stand independently. When she stood with support, her knees hyperextended, and her toes touched the ground. In addition, she could not pinch pellets with her thumb and index finger or eat with a spoon. For language development, she could only pronounce “mama” and “papa” unconsciously. Her vision and hearing were normal. The proband was given ganglioside and aceglutamide to treat nerve cell damage. Comprehensive rehabilitation training was given to the proband, including repetitive transcranial magnetic stimulation, occupational therapy, and cognitive therapy.

### Novel *CASK* mutation

**FIGURE 3:**
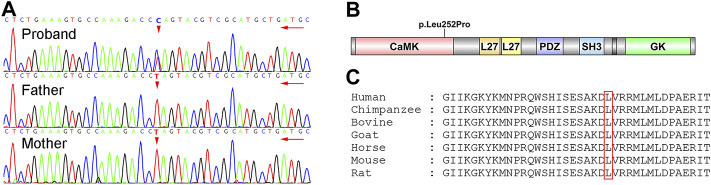
Identification of the novel *CASK* mutation. **(A)** results of Sanger sequencing. The proband was heterozygous status with c.755T>C mutation, but her parents were wild-type status. **(B)** functional domains of *CASK*. p.Leu252Pro mutation was in the CaMK domain of *CASK*. **(C)** conservation of the p.Leu252Pro mutation site in different species.

A novel heterozygote nonsynonymous mutation, NM_003688.3:exon8:c.755T>C:p.Leu252Pro in the *CASK* gene (gene position: chrX: 41660515) was identified in the proband (Figure 3), and the amino acid changed from leucine to proline. The influenced codon “L” was conserved. However, the *CASK* mutation was not identified in her parents after Sanger sequencing. According to the American College of Medical Genetics and Genomics (ACMG) guidelines, this *CASK* mutation was categorized as a pathogenic mutation (PS2+PM1+PM2+PP2+PP3) because: 1) this mutation was a novel mutation verified by the venous blood samples of her parents (PS2); 2) this mutation occurred in the CaMK functional domain of *CASK* protein (PM1); 3) this mutation was not found in the Berry big data population database, ExAC, 1000 Genome Project, or gnomAD (PM2); 4) a missense mutation of the *CASK* gene was the common mechanism for the phenotypes of related diseases, and the proportion of benign missense mutation was low (PP2); 5) the results of the in silico prediction algorithms (SIFT, Polyphen2, MutationTaster, REVEL, MCAP and LRT) showed that this *CASK* mutation had damaging or possibly damaging effects on genes or gene products, and the results of SPIDEX showed that the mutation might affect splicing (PP3).

## Discussion

The *CASK* protein is highly expressed in the central nervous system and engages in brain development and synaptic function. Intriguingly, the function of the *CASK* gene varies with the periods of brain development (Hsueh, 2006; Rivas et al., 2017). *CASK* mutations lead to interference of the neuronal migration and consequent neurodevelopmental disorder. Accumulated evidence (Hayashi et al., 2017; Nuovo et al., 2021) has demonstrated that most patients with MICPCH arise from *CASK* mutations. Although some reported maternally inherited *CASK* mutations, the majority were *de novo* (Seto et al., 2017; Pan et al., 2021). Most missense mutations cause alteration of protein structure through a frameshift change of amino acids, while some missense mutations cause misrecognition of specific sites by splicing machinery (Cartegni et al., 2002). The reduced accuracy of splicing yields aberrant transcripts that lead to less-functional protein and ultimate gene inactivation. Piluso et al. (2009) demonstrated that the missense mutation exon2:c.83G>T(p.R28L) in the *CASK* gene did not induce significant alterations in the structure, dynamics, and functions of *CASK*, but they found aberrant exon2-skipped *CASK* mRNA transcripts ascribed to the improper recognition of exonic splicing enhancers. In this study, we found a novel *CASK* mutation in the CaMK domain, and leucine (252nd amino acid) was replaced with proline. Neurexin and other molecules interact with the *CASK* protein through the CaMK domain. A missense mutation (p.Leu209Pro) was reported that could disrupt protein interactions mediated by the CaMK domain but not directly affect the *CASK*-neurexin interaction (LaConte et al., 2019). However, whether the neurexin binding function of *CASK* protein was affected by p.Leu252Pro mutation needs to be further explored.

Several studies have investigated the pattern of X-chromosome inactivation for mutations in the *CASK* gene. Burglen et al. (Burglen et al., 2012) performed an X-inactivation study on nine female patients with *CASK* anomalies and found that all of them had random X-chromosome inactivation. A random skewing X-inactivation pattern was also found in a 12-year-old female with a *CASK* mutation of c.1556T>C:p.Met519Thr (LaConte et al., 2018). However, in a family with a maternally inherited allele carrying a *CASK* mutation, the mother had the skewed X-chromosome inactivation, but the daughter had a paradoxical X-chromosome inactivation (Seto et al., 2017).

The phenotypes of mutations in the *CASK* gene are diverse. *CASK* null alleles lead to severe phenotypes, while missense mutations and slicing mutations in the *CASK* gene lead to mild to moderate phenotypes (Moog et al., 2011). The MRI images of a patient with MICPCH caused by *CASK* mutations often show prominent cerebellar hypoplasia, different degrees of pons hypoplasia, and a normal-sized corpus callosum, which might be important imaging clues for distinguishing the patients with *CASK* mutations from other MICPCH patients (Takanashi et al., 2010). Takanashi et al. (Takanashi et al., 2012) detected a normal to low-normal size corpus callosum in each of 16 patients with intellectual disability and MICPCH with *CASK* mutations. Our proband displayed cerebellar hypoplasia and bilateral enlarged brain ventricles, which was in line with another study (Zhao et al., 2021). Microcephaly, the key feature of MICPCH, could be determined by head circumference smaller than –3SD below the median (DeLuca et al., 2017). Zhao et al. (Zhao et al., 2021) observed four female patients with MICPCH caused by *CASK* mutations whose head circumference ranged from –4.2 SD to –7.7 SD. Hayashi et al. (Hayashi et al., 2012) described that the occipitofrontal circumferences of ten female patients with MICPCH were obviously lower than the normal standard (<–3.2 SD). In our study, the head circumference of the proband was initially within the normal range and nearly stagnant since the age of 3 months. *CASK* regulates mitochondrial respiration and oxidative metabolism, and nutrition and energy deprivation of the rapid growth of brain after birth disproportionately limits the growth of the cerebellum (Srivastava et al., 2016; Zhao et al., 2021). Moreover, the differences in symptoms and manifestations, especially in language and motor function, between patients with microcephaly and healthy children become more and more obvious during the developmental progression (DeLuca et al., 2017). This would explain the phenomenon that MICPCH patients tend to be born with normal head circumference, birth weight and length but experience postnatal progressive microcephaly and growth retardation. Pan et al. (Pan et al., 2021) reported that among six patients with novel *CASK* mutations, four patients had developmental delay or intellectual disability, and three patients had muscular hypertonia, hearing loss and ophthalmologic abnormalities. In ten females with *CASK* mutations, two patients had seizures, and five patients had muscular hypotonia (Hayashi et al., 2012). Due to the variable genotype-phenotype of *CASK*, the diseases arising from *CASK* mutations should be diagnosed with caution.

## Conclusion

Collectively, this study reported a novel c.755T>C(p.Leu252Pro) *CASK* mutation in a female Chinese child with MICPCH, which might broaden the spectrum of *CASK* genotype and is of great value for precise prenatal diagnosis and genetic counseling.

## Data Availability

The datasets presented in this study can be found in online repositories. The names of the repository/repositories and accession number(s) can be found below: https://www.ncbi.nlm.nih.gov/, SRR17634755 https://www.ncbi.nlm.nih.gov/, SRR17634754 https://www.ncbi.nlm.nih.gov/, SRR17634753.
